# High-resolution quantitative spiral perfusion for microvascular coronary dysfunction detection

**DOI:** 10.1186/1532-429X-16-S1-P227

**Published:** 2014-01-16

**Authors:** Yang Yang, Sujith Kuruvilla, Craig H Meyer, Frederick H Epstein, Angela M Taylor, Christopher M Kramer, Michael Salerno

**Affiliations:** 1 Biomedical Engineering, University of Virginia, Charlottesville, Virginia, USA; 2 Medicine, University of Virginia, Charlottesville, Virginia, USA; 3 Radiology, University of Virginia, Charlottesville, Virginia, USA

## Background

Quantitative first-pass CMR can assess the severity of microvascular coronary dysfunction(MCD) in subjects with signs and symptoms of ischemia but without obstructive coronary artery disease(CAD). In patients with MCD, abnormalities of myocardial perfusion reserve(MPR) occur globally but may also vary in their transmural extent. Thus, high spatial resolution quantitative CMR perfusion imaging is needed to differentiate between the subendocardial and subepicardial layers. Given the efficiency and SNR of spiral trajectories, we have developed the accelerated high-resolution absolute quantification spiral pulse sequence to assess variations in the transmural extent of myocardial perfusion abnormalities in patients with suspected MCD.

## Methods

We developed an accelerated high-resolution perfusion pulse sequence based on our previous dual contrast quantitative spiral sequence. Sequence parameters included: 8 interleaves of variable density spirals from 0.75 to 0.2 Nyquist, 6.1 ms readout per interleaf, TE 1.0 ms, TR 9 ms, TI 80 ms, FA 35°, FOV 320 mm^2^, in-plane resolution 1.5 mm. Resting perfusion were collected in 5 volunteers and adenosine stress CMR was performed in one suspected MCD patient. Perfusion images were acquired at 3 short axis slice locations on a 1.5T Siemens Avanto scanner during injection of 0.1 mmol/kg of Gd-DTPA. Stress imaging was performed following a 4 minute infusion of adenosine(140 mcg/kg/min). Quantification of perfusion was performed on a pixel-wise basis using Fermi-function deconvolution after image was reconstructed by SPIRiT and aligned with non-rigid registration ANTS.

## Results

**Figure 1:**
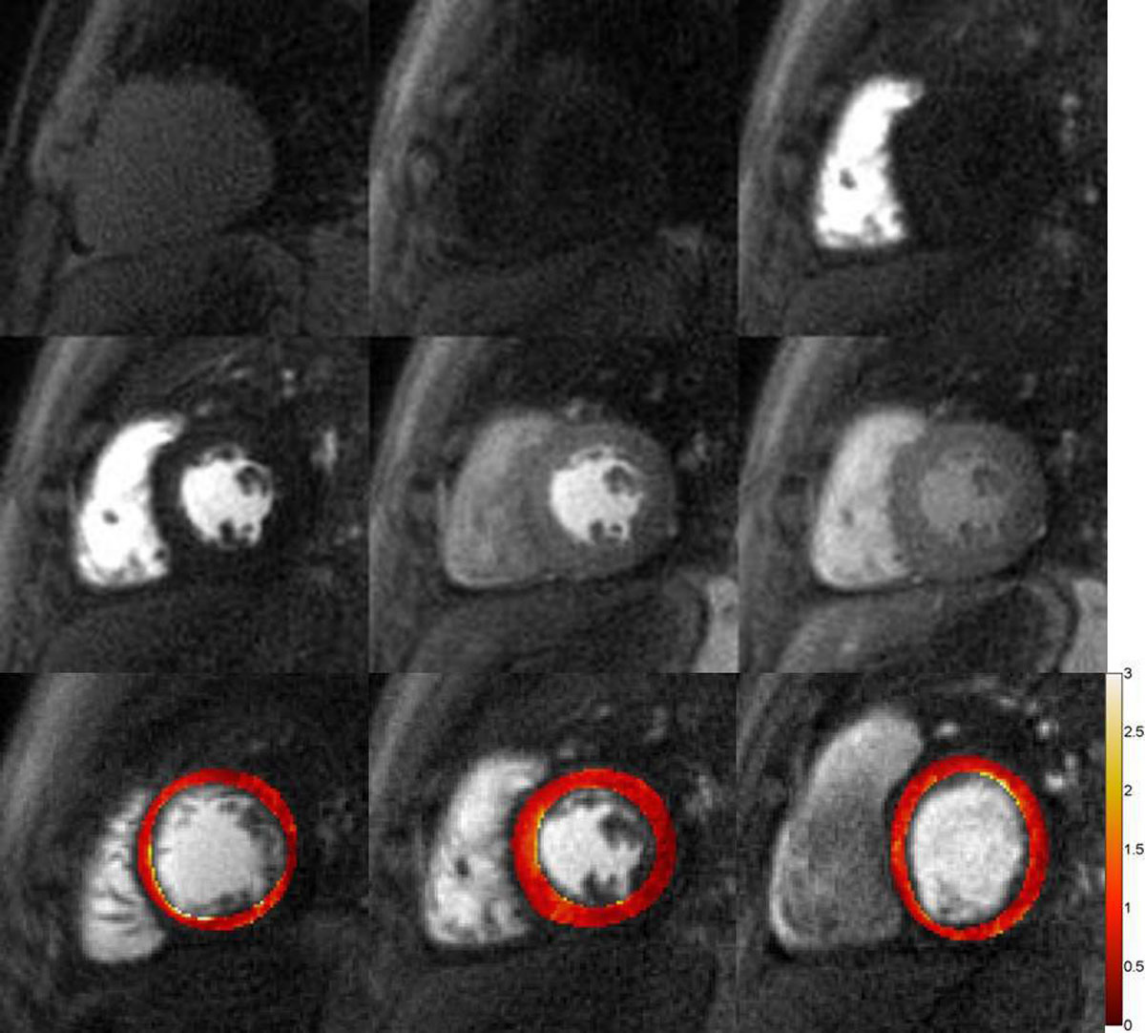
**First 2 rows: Reconstructed proton density image and perfusion images at different time point from one healthy volunteer**. Last row: Myocardial blood flow maps at rest

**Figure 2:**
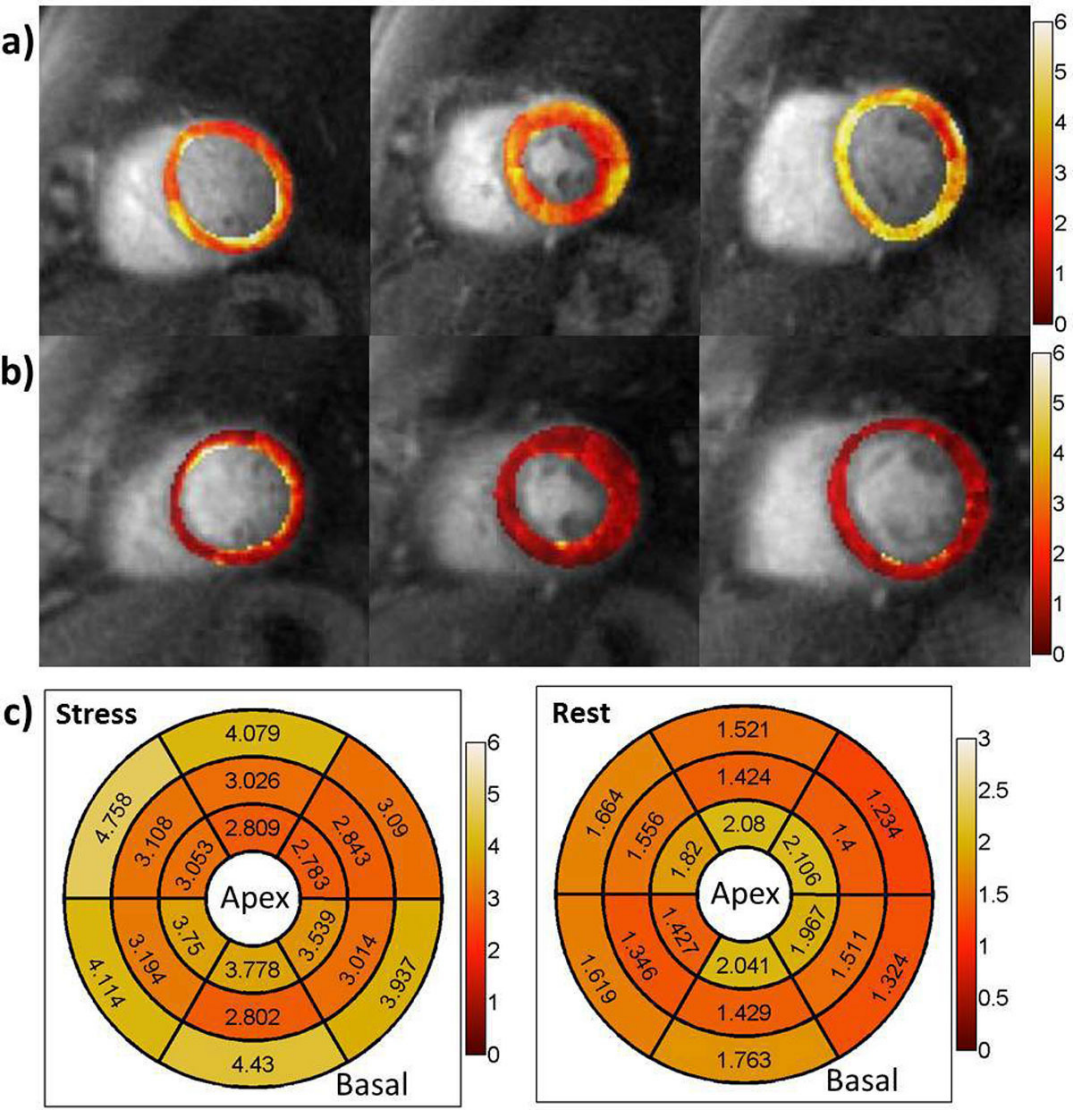
**Pixel-wise MBF maps at stress (a) and rest (b), as well as the segment MBF results (c) from the patient with suspected MCD**.

Figure 1 shows the reconstructed high-res perfusion images and the myocardial blood flow (MBF) map from one volunteer at rest. The first two rows are SPIRiT reconstructed and registered proton density image and perfusion images at different time point from the same slice. The images have adequate SNR despite the high in-plane resolution of 1.5 mm. Furthermore as the reconstruction does not rely on temporal correlations in the data, the sequence is robust to motion during acquisition. The last row shows the uniform MBF map from all the 3 slices. Figure 2 shows pixel-wise maps of MBF at stress(a) and rest(b), as well as the segment MBF results(c) from the subject with suspected MCD. This subject shows the normal increase in subepicardial perfusion but reduced perfusion to the subendocardium following adenosine infusion. The global MRP is reduced(2.17). Also in the middle slice, the subendocardial MBF is 2.69 ± 0.28 mL/g/min, while the subepicardial MBF is 3.25 ± 0.26 mL/g/min.

## Conclusions

We demonstrate the successful application of high-resolution quantitative spiral perfusion sequence in both healthy volunteers and in a patient with suspected MCD. This technique provides high quality images and high resolution MBF to further investigate the transmural variation of MBF in patients with MCD and evaluate the prognostic significance of abnormal perfusion in these MCD patient subsets.

## Funding

K23 HL112910, R01 HL79110

